# Development of Hybrid and Templated Silica-P123 Membranes for Brackish Water Desalination

**DOI:** 10.3390/polym12112644

**Published:** 2020-11-10

**Authors:** Muthia Elma, Dwi Rasy Mujiyanti, Noor Maizura Ismail, Muhammad Roil Bilad, Aulia Rahma, Sazila Karina Rahman, Fitriani Fitriani, Arief Rakhman, Erdina Lulu Atika Rampun

**Affiliations:** 1 Chemical Engineering Department, Faculty of Engineering, Lambung Mangkurat University, Banjarbaru, South Kalimantan 70714, Indonesia; karina.sazila@gmail.com; 2 Materials and Membranes Research Group (M2ReG), Lambung Mangkurat University, Banjarbaru, South Kalimantan 70714, Indonesia; arahma@mhs.ulm.ac.id (A.R.); fitrianimihardjo@gmail.com (F.F.); arf.rkhmn@gmail.com (A.R.); elarampun@mhs.ulm.ac.id (E.L.A.R.); 3 Chemistry Department, Faculty of Earth Science, Lambung Mangkurat University, Banjarbaru, South Kalimantan 70714, Indonesia; drmujianty@ulm.ac.id; 4 Faculty of Engineering, Universiti Malaysia Sabah, Jln UMS, Kota Kinabalu 88400, Sabah, Malaysia; 5 Department of Chemical Engineering, Universiti Teknologi PETRONAS, Bandar Seri Iskandar 32610, Perak, Malaysia; mroil.bilad@utp.edu.my

**Keywords:** mesoporous silica, rapid thermal processing, sol–gel process, brackish water desalination

## Abstract

Water scarcity is still a pressing issue in many regions. The application of membrane technology through water desalination to convert brackish to potable water is a promising technology to solve this issue. This study compared the performance of templated TEOS-P123 and ES40-P123 hybrid membranes for brackish water desalination. The membranes were prepared by the sol–gel method by employing tetraethyl orthosilicate (TEOS) for the carbon-templated silica (soft template) and ethyl silicate (ES40) for the hybrid organo-silica. Both sols were templated by adding 35 wt.% of pluronic triblock copolymer (P123) as the carbon source. The silica-templated sols were dip-coated onto alumina support (four layers) and were calcined by using the RTP (rapid thermal processing) method. The prepared membranes were tested using pervaporation set up at room temperature (~25 °C) using brackish water (0.3 and 1 wt.%) as the feed. It was found that the hybrid membrane exhibited the highest specific surface area (6.72 m^2^·g^−1^), pore size (3.67 nm), and pore volume (0.45 cm^3^·g^−1^). The hybrid ES40-P123 was twice thicker (2 μm) than TEOS-P123-templated membranes (1 μm). Lastly, the hybrid ES40-P123 displayed highest water flux of 6.2 kg·m^−2^·h^−1^. Both membranes showed excellent robustness and salt rejections of >99%.

## 1. Introduction

Brackish water is one of the major problems faced in mining areas, and worsens when pollutant pollutes the freshwater resources [1]. There are several membrane processes that are promising for treatment of brackish water, namely, forward osmosis [2], reverse osmosis [3], membrane distillation [4], and pervaporation [5]. Among them, desalination by the pervaporation process is particularly attractive because it employs low pressure but still results in high water flux. Pervaporation allows the separation of a mixture using a membrane (nonporous or porous) through partial vaporization [6].

Selection of membrane in pervaporation is very crucial, particularly on the material building block and the pore size. Microporous material, for example, is usually applied for gas separation [7,8,9,10], but is not suitable for water desalination because of the poor water flux. Mesoporous material is more attractive in pervaporation [11,12]. Mesoporous material has a larger pore size, ranging from 2–50 nm, and offers higher water flux. The use of a dual acid-base catalyst is the most important key to control this pore size. Mesoporous silica gains attention because of a thermally stable and strong structure [13,14]. Silica has unique physiochemical properties composed of a cross-linked network structure [15,16] and vigorous [17] and high surface area [18].

Despite these excellent characteristics, silica has limitations in water desalination applications. Silanol groups (Si–OH) in silica may affect pore enlargement when associated with water via hydrogen bonding [19]. It leads to poor membrane performance in spite of offering high water flux or salt rejection. Few examples of silica-based membrane materials include inorganic-organic hybrid nanocomposite materials [20], carbon-templated silica, and metal oxide (nickel [21,22,23], cobalt [24,25,26], and iron [7]). An inorganic-organic hybrid nanocomposite and carbon-templated silica can practically be produced by using a sol–gel process. Condensation in the sol–gel process allows organic polymer or polymer to bind with inorganic network [27,28], while, in carbon template, the carbon is added after the silica sol was produced [29,30]. Inorganic-organic hybrid nanocomposite materials and carbon-templated silica are a promising polymer membrane modified version for pervaporation process through water desalination. This modified version can improve the membrane performance such as increase the selectivity, salt rejection, resistance to fouling, and good stability and save production time and cost. Based on our previous works [31,32,33], the modified hybrid and templated membrane are very suitable to separate salt molecules and allow water molecules to pass through the membrane matrices. In addition to providing strength to the membrane pores, the addition of P123 in silica sol affects the silanol and siloxane groups in the silica structures. Silanol and siloxane groups are known to affect pore size of the membrane [32].

Various silica membrane precursors have been developed, including tetraethyl orthosilicate-(TEOS), tetra ethyl vynilsilane (TEVS), bis(triethoxysilyl)ethane (BTESE), methyltriethoxysilane (MTES), [34] and ethyl silicate-40 (ES40) [14,35]. These precursors are mainly found in an inorganic, multipurpose chemical compound within quartz, sand, or flint [36]. The most common precursor is TEOS because it acts differently under similar conditions [37].

Another important aspect in pervaporation membrane fabrication is the calcination process. It can be categorized into two types: The conventional thermal processing (CTP) and the rapid thermal processing (RTP). The RTP does not need dwelling time, unlike the CTP (4 h with 1 °C min^−1^) [38]. However, membrane preparation using the RTP method is faster than the CTP, which typically requires 10–14 days of duration [39,40,41,42,43].

Many previous researchers have used the TEOS [29,44], but only a few researchers employed ES40 [13]. Membrane usually also consists of thin film, -Al_2_O_3_ interlayer with pore size of >0.05 m, and substrate of >0.5 m [45]. In this work, thinner membranes were developed by direct coating of sols onto the substrates in a process called interlayer-free membrane. Herein, we fabricated and evaluated the performance of the interlayer free of silica-P123 membrane by using hybrid (ES40) and template (TEOS) for pervaporation of brackish wastewater. P123 was chosen as the carbon because it can increase hydro-stability of silica. The membranes were calcined by using the RTP method and characterized via Brunner–Emmet–Teller (BET).

## 2. Materials and Methods 

### 2.1. Sol–Gel Synthesis and Characterization

A carbon-templated silica sol was made through the following process. Firstly, TEOS (99.0%, Sigma-Aldrich, St. Louis, MO, USA) was mixed with ethanol (EtOH, 70%) and stirred at 0 °C for 5 min. Nitric acid solution (0.0008 M HNO_3_, Merck, Kenilworth, NJ, USA) was then added dropwise and refluxed at 50 °C for 1 h. Ammonia (0.0003 M NH_3_, Merck) was then added into the solution and stirred for 2 h. The final sol was tested until pH ~6. Afterwards, the prepared silica sol was templated by adding P123 (35 wt.%, Sigma-Aldrich) and stirred for 45 min at room temperature (~25 °C). The final molar ratios of the carbon-templated silica (TEOS-P123) sol of TEOS:EtOH:HNO_3_:H_2_O:NH_3_:P123 was calculated to be 1:38:0.0008:5:0.0003:0.00024. The P123 concentration of 35 wt.% was chosen based on our previous research, which prepared membrane using vacuum condition and CTP calcination

Meanwhile, the hybrid ES40-P123 sols were prepared by adding ES40 (Indonesian Chemicals of PT. Grasindo Multi Sentosa) into the ethanol. The solution was stirred for 5 min at 0 °C. During the stirring process, nitric acid (0.00078 M HNO_3_, Merck) was added dropwise into the solution and was refluxed for one hour at 50 °C. Then, ammonia (0.00003 M NH_3_, Merck) and P123 (35 wt.%, Sigma-Aldrich) were mixed into the solution, and refluxed for 2 h under the same condition. Similar to the carbon-templated silica sol, the pH of this sol was measured until it showed a pH of ~6. The process resulted in a molar ratio of ES40:EtOH:HNO_3_:H_2_O:NH_3_:P123 to be 1:120:0.0022:16:0.009:0.1.

Both the hybrid and the templated silica sol were dried in the oven for 24 h at 70 °C. After that, the dry sols were grounded into powder to form xerogels. Later, the xerogels were calcinated at 350 °C in a furnace for 1 h using the RTP method. The pore properties of the xerogels were characterized by Nitrogen physisorption analysis at 77 K and 1 bar was conducted using Micromeritic TriStar 3000 instrument of xerogel was degassed at 200 °C for >6 h under vacuum condition. The specific surface area was decided using Brunner–Emmet–Teller (BET) method. The average pore sizes of microporous and mesoporous materials were taken by Dubinin–Astakhov and Barrett–Joyner–Helenda methods, respectively.

### 2.2. Membrane Fabrication and Testing

**Figure 1:**
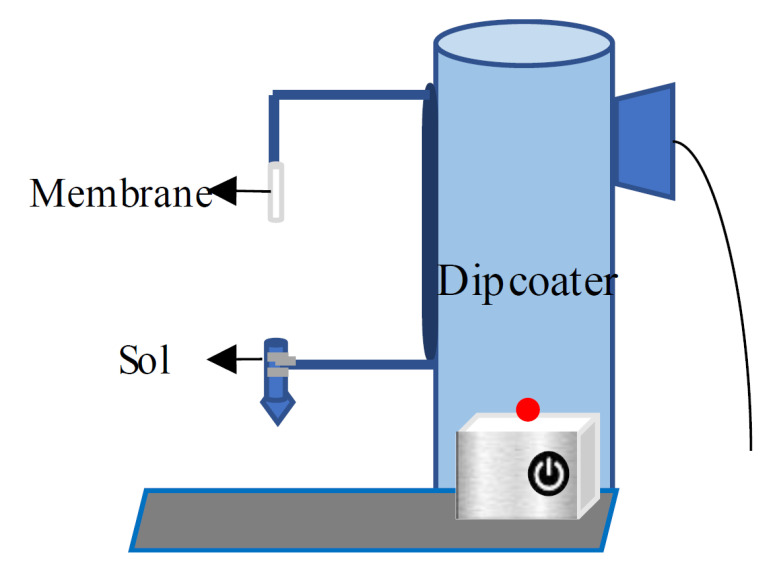
Dipcoater setup of silica thin film preparation.

The hybrid and the templated silica sols were dip-coated directly onto microporous alumina substrates’ α-Al_2_O_3_ tubular support (Ceramic Oxide Fabricators, California Gully, Australia) (Figure 1). The membrane was calcined at 350 °C for 1 h using the RTP method. The coated support was cooled at room temperature. The dip-calcine-cooling process was repeated for four times to produce the four coated layers. The membrane morphology and thickness were inspected using scanning electron microscopy (SEM ZEISS, Oberkochen, Germany).

**Figure 2:**
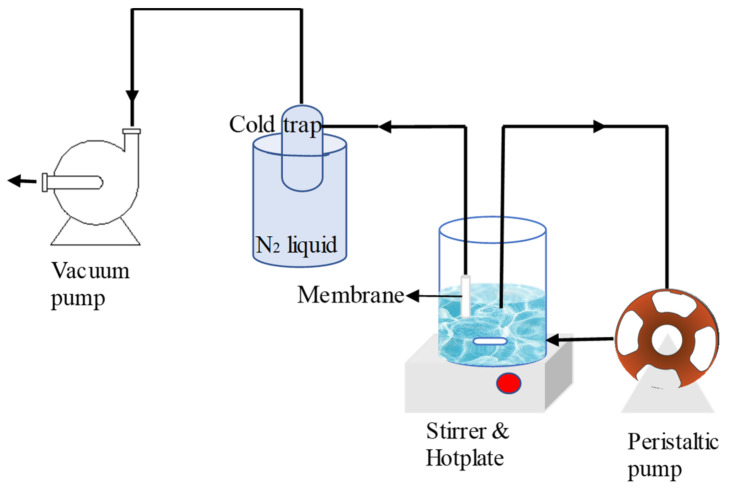
Membrane desalination using a customised pervaporation setup.

Membrane pervaporation performance was measured by using the pervaporation test illustrated in Figure 2. The membrane was exposed in feeds of demineralized water and brackish water (0.3 and 1 wt.%) placed in a large beaker (1 L). The inner membrane filtration cell was connected to the vacuum pump (<1 pa). The brackish feed water was prepared by using NaCl (Sigma-Aldrich) (0.3–1 wt.%). During the test, the feed was stirred to prevent salt buildup on the outer shell of the membrane. The permeate was collected in the cold trap by using the liquid nitrogen as condenser. The water flux (F) was determined using Equation (1):

(1) $$F=m/\left(A∆t\right)$$

where *m* is a permeate mass (kg) collected in the cold trap, *A* is the surface area (m^2^) of membrane, and *Δt* is time operation (h). Meanwhile, the salt rejection (*R*) was calculated by using Equation (2).

(2) $$R=\frac{C_{f}-C_{p}}{C_{f}}\times 100\%,$$

where *C_f_* and *C_p_* are the salt concentration of the feed and the permeate (wt.%), both measured by a conductivity meter (OHAUS).

## 3. Results and Discussion

### 3.1. Membrane Characteristics

**Figure 3:**
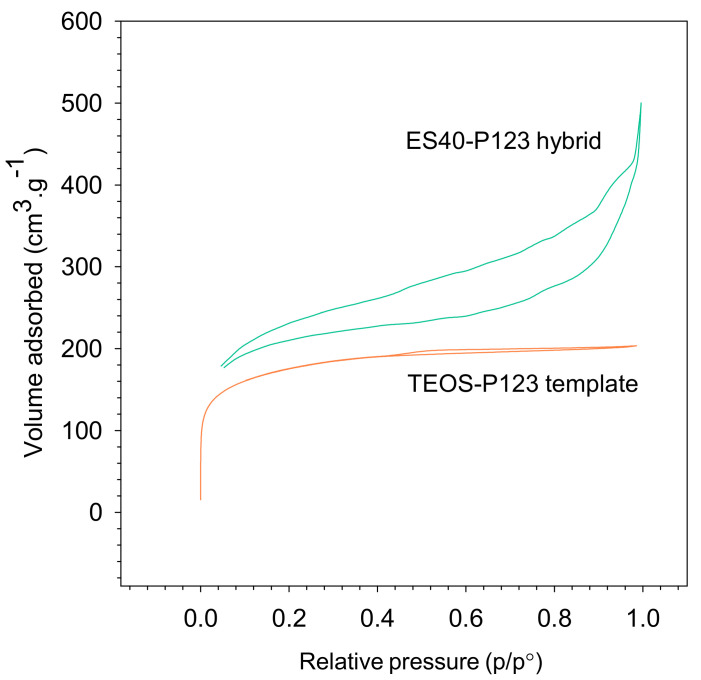
BET adsorption of carbon-templated silica (P123-TEOS) and hybrid organosilica (P123-ES40).

BET adsorptions of carbon-templated silica (P123-TEOS) and hybrid organosilica (P123-ES40) are shown in Figure 3. It shows a type-IV isotherms’ hysteresis for both the carbon-templated silica (P123-TEOS) and the hybrid organosilica (ES40-P123), indicating the characteristic of a mesoporous structure. A similar result was also found in the silica membrane using TEOS or ES40 [39]. However, the finding disagrees with the earlier report in which the carbon 35 wt.% P123-templated silica (TEOS) using the CTP method calcined in N_2_ and vacuum exhibited the type-I isotherm without any hysteresis. The P123 micelles in a solution strongly affected the mesostructure. Increasing P123 incorporation increased the mesoporosity, too. In addition, the use of the RTP method for the ES40 membrane fabrication resulted in a more porous structure compared to the dense membrane calcined in CTP [39]. Besides the calcination method, the condition held in air, vacuum, or gases (N_2_, CO_2_, CH_4_, etc.) also affected the structure of the material. The decomposition of carbon was much easier under the atmospheric condition. 

A study using a hard template method also resulted in material with type-II b isotherm under a low mass ratio of chitosan(carbon)/silica. In our study, TEOS-P123 templated was prepared using the soft template method involving the interaction between inorganic and surfactant via covalent bonds, electrostatic forces, or hydrogen bonding [46]. A hard template is often used because it offers a good stability and better control on the resulting product properties. Nevertheless, the template removal procedure may cause a serious environmental problem owing to the use of harmful etchants as the remover [47]. 

Hysteresis, as observed in Figure 3, corresponds to a capillary condensation in mesoporous structure. There are six types of hysteresis: H1, H2(a), H2(b), H3, H4, and H5, according to the IUPAC committee classification of 2015. The all characteristic hysteresis types is rather closely related to particular features of the pore structure and underlying adsorption mechanism. The IUPAC classification of adsorption isotherms types The type of hysteresis loop for TEOS-P123 templated is H4 with relative pressure of about 0.4–0.8. It has narrow, slit-like pores and is nearly horizontal [48]. It was also observed that the ES40-P123 hybrid resulted in a type of hysteresis loop of H3 with a relative pressure of 0.05–0.9. The branch had a slope in a large range of relative pressure and resembled the type-II isotherm. This type of hysteresis indicates the existence of macropores in a sample that is incompletely filled with pore condensate. Another report on the carbon–silica composite material showed the type H2 hysteresis loop [49]. It is worth noting that in their work they used TEOS with the addition of silica material and activated carbon powder and applied a weak base catalyst (NH_4_OH) through the sol–gel method. In our study, we employed both a strong acid and strong base catalysts. The strong acid catalyst promoted more micropore with an average pore size of less than 2 nm and end-of-chain condensation, while the base catalyst contributed to the mesoporous size (an average pore size in the range 2–50 nm). However, in a recent study, a mesopore can be formed, too, by applying weak acid (citric acid) catalyst [44,50].

The data for BET surface area, pore volume, and average pore diameter for both samples are summarized in Table 1, in which both membranes are mesoporous. The hybrid organosilica showed the highest values of the surface area, pore volume, and average pore diameter of 671 m^2^·g^−1^, 0.45 cm^3^·g^−1^, 3.67 nm, respectively, which resulted in a higher water flux, as discussed later. It indicates that more siloxane was formed over silanol during the reaction, as reported by others, including Mrowiec-Bialon, et al. [51]. The condensation is favored over the hydrolysis reactions. Larger surface area and pore volume were also reported for the heteropolyacid-silica composite catalyst for ES40 [52]. A greater degree of cross-linking and a more steric hindrance effect promoted ES40 to have a larger porosity and inhibited densification throughout the thermal process, resulting in a more open silica network. Silanol reduction showed the potential of ES40 in improving hydrostability. The cost was also lower compared to TEOS precursor [35].

Another important factor affecting the properties is the slower hydrolysis reaction of the sol–gel process under low temperatures. It subsequently slowed down the condensation reaction, as suggested elsewhere [39]. This behaviour was dissimilar to the TEOS sol–gel system [53]. ES40 is a partly condensed TEOS oligomer consisting of 40 wt.% (trimeric and tetrameric) of the silica. A hydrolysis rate of ethyl silicate should be under control to yield high surface area because the growth of large particles was restricted by the trimeric and the tetrameric silica species [54].

**Figure 4:**
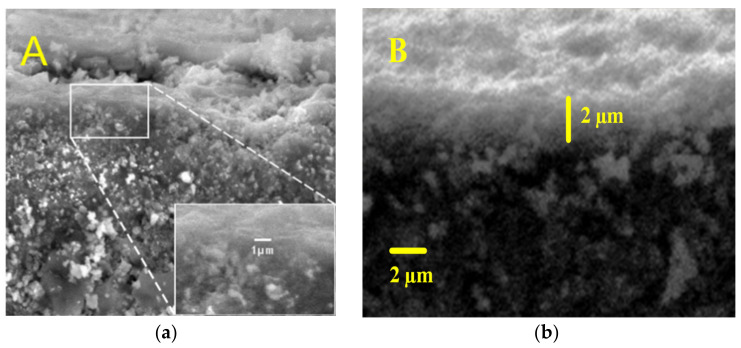
SEM images respective to (**a**) carbon-template silica vs. (**b**) hybrid silica of membranes’ thickness.

Figure 4 shows the cross sections of carbon-templated silica and hybrid organosilica, with two layers consisting of a macroporous α-Al_2_O_3_ substrate at the bottom and a thin layer on the top. Both membranes have rough cross-section morphologies and smooth surfaces. The microstructure images suggest that a homogeneous sol could provide a decent flux and rejection, as proven later. It results in a crack-free membrane. The top layer thicknesses of both membranes are 1 and 2 μm for the carbon-templated silica and the hybrid organosilica, respectively, which can be ascribed to the structural differences. ES40 has a higher degree of condensation than TEOS and would typically undergo cluster–cluster growth with siloxane bridges. Here, both membranes were prepared by the RTP method, which was thicker than membranes fabricated using CTP in a previous study (470 nm). The RTP increased the condensation and did not employ the ramping rates, resulting in a very fast solvent evaporation during the sudden rise of calcination.

### 3.2. Membrane Pervaporation

**Figure 5:**
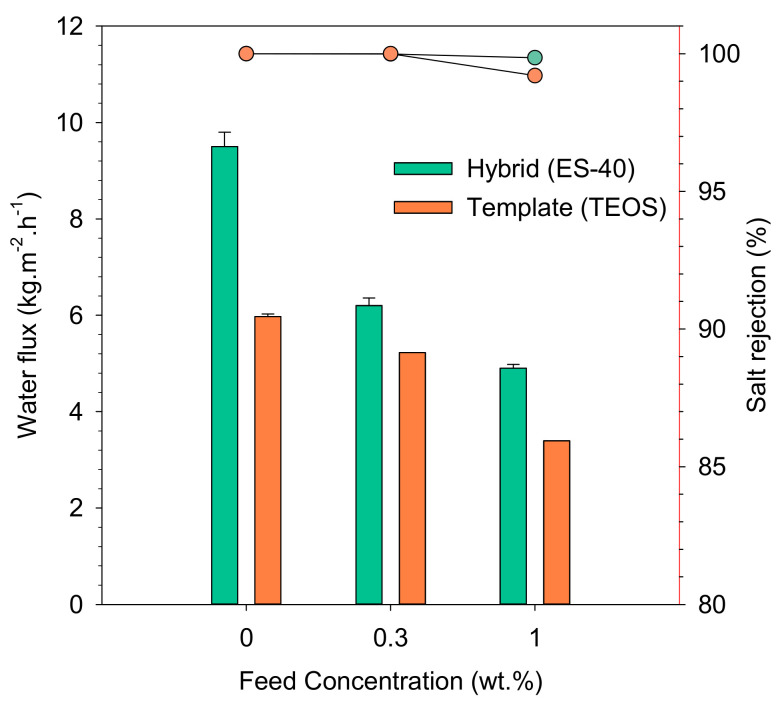
Pervaporation fluxes and salt rejections of hybrid (ES40) and templated (TEOS) membranes for brackish water filtration.

Figure 5 represents the performance of both membranes for pervaporation. Aquadest and brackish (0.3–1%) feeds’ pervaporation were performed at room temperature (25 °C) through hybrid organosilica (ES40-P123) or carbon-template silica (TEOS-P123). ES40-P123 hybrid presented the highest water flux for all feeds. It exhibited water flux of 9.5 kg·m^−2^·h^−1^ for aquadest and 6.2 kg·m^−2^·h^−1^ for the brackish water feed. All the membranes showed good salt rejections of >99%. This result is clearly attributed to the highest surface area, pore volume, and average pore diameter of the hybrid organosilica (Table 1).

Figure 5 also shows that the drop of water fluxes along with the increasing salt concentration occurred because of the salt polarization phenomena and pore blocking (Figure 5). This finding was in line with a previous study [57]. Salt concentration near the membrane surface increased due to evaporation, resulting in depletion of the driving force of the permeate. Larger salt ions would be blocked by percolative pathways and would also reduce the water fluxes.

A suitable catalyst was chosen based on the desired pore properties. In this work, a dual acid-base catalyst with pH 6 contributed to the percolative pathway formed to constrict to smaller than the size of Na^+^ (7.2 Å) and Cl^−^ (6.6 Å) but larger than water molecule’s size (2.6 Å), also called the bottleneck pore. This range of pore size is ideal for water desalination, but too large for gas separation. Gas separation mostly uses smaller membrane pores, achieved by employing an acid catalyst [7].

Hybrid organosilica membrane demonstrated excellent water fluxes due to the strong chemical covalent bonds between ES40 and P123. The incorporation of P123 (as an organic group) was achieved by bonding to the silicon moieties. It introduced organic (P123) bridges to the inorganic network (ES40) structure. This combined structure has been suggested to inflict a higher toughness of the material [58].

**Figure 6:**
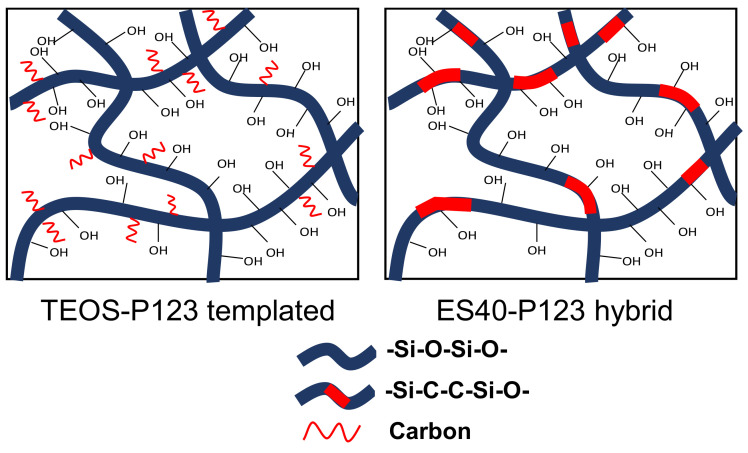
Schematic of P123 template silica (TEOS) and hybrid P123 (ES40) structure.

Hybrid material structure was combined in a single solid both of properties with mechanically and thermally stable backbone and chemical reactivity, as well as the flexibility of the organo-functional group [59]. They were mostly cross-linked, and an improvement would be achieved when the siloxane bridges (Si–O–Si) in ES40 are replaced by the organic bridges (Si–C–C–Si) from the carbon of P123. In this study, we also employed a noncovalent linkage of P123 and TEOS, as illustrated in Figure 6. This idea emerged after some considerations. First, TEOS was preferred as the source of the silica material because it is easier to control. Second, P123 has been known as a carbon precursor that further increases the pore volume and surface area in silica. In the template membrane case, carbon from P123 did not replace the inorganic bridge. Both of membrane-modified techniques resulted in a better hydro-stability.

Recently, interlayer-free membrane has been invented to address the effect of thickness on the membrane performance [25,41]. Reducing the thickness would improve the water fluxes and lower the water resistance. Many membranes are thus composed of (1) support, which is designed to establish mechanical robustness, (2) interlayer or middle layer, which acts to hinder the way of any particles to enter into the pores of the underlying support layer and makes the top layer surface more smooth because the solution does not directly infiltrate into the substrate, and (3) top layer, which is a thin film governing the selectivity [45].

Data in Table 2 show that the carbon silica membrane poses better mechanical strength than the unmodified silica. It can be deduced from the higher water flux and salt rejection in various salt concentrations. Carbon addition into silica using the template or hybrid prevents the pores from collapsing. Both membranes showed an excellent performance, whether it was made via the RTP or the CTP calcination methods. An earlier study reported that the CTP provides higher surface area than the RTP. Yet, RTP did not significantly affect the membrane performance. The water fluxes and salt rejection were still high because they had similar pore size and pore volume [38]. Moreover, the RTP method saved cost and time during the membrane fabrication.

Table 2 shows a comparison of the hybrid and template membrane created in air vs. vacuum under various feed temperatures. It demonstrates that high water fluxes and good salt rejection were achieved for membranes calcined in air condition. The calcination in air still allowed the prepared silica membrane to contain more siloxane and silica-carbon bonds, despite the fact that partial decomposition of carbon under air was higher than under vacuum [38]. Table 2 also shows that pervaporation membranes reached a high level of water fluxes as well as salt rejection under different feed temperatures and salt concentrations, quite competitive with the reverse osmosis. Reverse osmosis requires much higher pressure (60–75 bar) and could not handle high salt concentration [60], while pervaporation can work under a pressure as low as 1 bar.

## 4. Conclusions

Hybrid organosilica and carbon-template silica membranes with controllable pore size were synthesized through the sol–gel process using ES40 and TEOS as silica precursors, respectively. P123 was used as an organic carbon material to modify the silica membrane. The results showed that the hybrid membrane exhibited a larger specific surface area of 6.72 m^2^·g^−1^, higher pore size of 3.67 nm, and a larger pore volume of 0.45 cm^3^·g^−1^. The hybrid organosilica was also thicker than the template membrane. The addition of P123 enhanced both membranes’ robustness. The combination of these properties resulted in a hybrid organosilica (ES40-P123) with the highest water flux of 9.5 kg·m^−2^·h^−1^ for deionized water and 6.2 kg·m^−2^·h^−1^ for the brackish water. Both membranes showed a good salt rejection of >99%. However, a major limitation on long-term stability of hybrid and templated membrane modification is membrane fouling. This resulted in decreased permeation flux, change in selectivity and separation during the filtration operation, and reduced membrane life, although the rejection of both membrane modifications gave good performance. Furthermore, this work paves the way for the development of sol–gel method using ES40 and TEOS combination as precursor silica membranes that further can be modified by being embedded with another inorganic or organic carbon content during membrane fabrication for water.

## Figures and Tables

**Table 1:** Surface area, pore volume, and pore diameter of TEOS-P123 templated and ES40-P123 hybrid, both calcined in air at 350 °C.

Membrane Type	BET Surface Area (m^2^·g^−1^)	Pore Volume (cm^3^·g^−1^)	Average Pore Diameter (nm)	Reference
TEOS-P123 templated	572	0.315	2.21	This work
ES40-P123 hybrid	671	0.45	3.67	This work
Carbonized silica membranes C16	793	0.37	-	[19]
Hybrid membrane	554	0.38	2.71	[55]
Carbonized C12 silica template	661	0.37	-	[19]
Carbonised P123-silica template	965	0.50	2.32	
Hybrid TEVS-P123	922	0.97	2.10	[56]

**Table 2:** Summary of studies on silica membrane for pervaporation.

Membrane Type	Calcination Method	Feed Temperatures (°C)	NaCl Concentration (%)	Water Flux (kg·m^−2^·h^−1^)	Salt Rejection (%)	References
**TEOS-P123 templated**	RTP (air)	25	0.3–1	5.2–3.4	>99.2	This work
**ES40-P123 hybrid**	RTP (air)	25	0.3–1	6.2–4.9	>99.8	This work
Carbonized silica membranes	CTP (air)	20	0.3–3.5	2.1–1.9	99.5	[58]
Carbonized silica membranes	CTP (vacuum)	20	0.3–3.5	3.2–1.4	89	[19]
Silica-pectin membranes	RTP (air)	25	3.5	5.73	>99	
Silica-P123 membranes	RTP (air)	25	3.5	1.49	99.8	
Silica membrane	RTP (air)	26	0.3–15	2.9–1.2	>95	[17]
Hybrid membrane	CTP (vacuum)	60–25	1–15	5.7–2.3	>99.7	[55]
Carbonised P123-silica template	CTP (vacuum)	22	0.3	1.7	>99.5	
Carbonised C16 silica template	CTP (vacuum)	60	0.3–3.5	3.1–2.1	91–97%	[19]
Hybrid TEVS-P123	CTP (vacuum)	60	0.3	3.7	>95	[56]
Hybrid organo-silica	CTP (air)	60–22	0.3–7.5	21–2	>98	

## References

[1] Rich V.I., Maier R.M., Pepper I.L., Gerba C.P., Gentry T.J. (2015). Chapter 6—Aquatic Environments. Environmental Microbiology.

[2] Nawi N.I.M., Arifin S.N.H.M., Hizam S.M., Rampun E.L.A., Bilad M.R., Elma M., Khan A.L., Wibisono Y., Jaafar J. (2020). Chlorella vulgaris broth harvesting via standalone forward osmosis using seawater draw solution. Bioresour. Technol. Rep..

[3] Choi W., Jeon S., Kwon S.J., Park H., Park Y.-I., Nam S.-E., Lee P.S., Lee J.S., Choi J., Hong S. (2017). Thin film composite reverse osmosis membranes prepared via layered interfacial polymerization. J. Membr. Sci..

[4] Alklaibi A.M., Lior N. (2005). Membrane-distillation desalination: Status and potential. Desalination.

[5] Elma M., Pratiwi A.E., Rahma A., Rampun E.L.A., Handayani N. (2020). The Performance of Membranes Interlayer-Free Silica-Pectin Templated for Seawater Desalination via Pervaporation Operated at High Temperature of Feed Solution. Mater. Sci. Forum.

[6] Van der Bruggen B., Luis P., Tarleton S. (2015). Chapter Four—Pervaporation. Progress in Filtration and Separation.

[7] Darmawan A., Motuzas J., Smart S., Julbe A., Diniz da Costa J.C. (2015). Binary iron cobalt oxide silica membrane for gas separation. J. Membr. Sci..

[8] Wang D.K., Diniz da Costa J.C., Smart S. (2014). Development of rapid thermal processing of tubular cobalt oxide silica membranes for gas separations. J. Membr. Sci..

[9] Miller C.R., Wang D.K., Smart S., Da Costa J.C.D. (2013). Reversible redox effect on gas permeation of cobalt doped ethoxy polysiloxane (ES40) membranes. Sci. Rep..

[10] Liu L., Wang D.K., Martens D.L., Smart S., Diniz da Costa J.C. (2015). Binary gas mixture and hydrothermal stability investigation of cobalt silica membranes. J. Membr. Sci..

[11] Liu L., Wang D.K., Kappen P., Martens D.L., Smart S., Diniz da Costa J.C. (2015). Hydrothermal stability investigation of micro- and mesoporous silica containing long-range ordered cobalt oxide clusters by XAS. Phys. Chem. Chem. Phys..

[12] Liu L., Wang D.K., Martens D.L., Smart S., Strounina E., Diniz da Costa J.C. (2014). Physicochemical characterisation and hydrothermal stability investigation of cobalt-incorporated silica xerogels. Rsc Adv..

[13] Rahman S.K., Maimunawaro, Rahma A., Syauqiah I., Elma M. (2020). Functionalization of hybrid organosilica based membranes for water desalination – Preparation using Ethyl Silicate 40 and P123. Mater. Today Proc..

[14] Maimunawaro, Karina Rahman S., Lulu Atika Rampun E., Rahma A., Elma M. (2020). Deconvolution of carbon silica templated thin film using ES40 and P123 via rapid thermal processing method. Mater. Today Proc..

[15] Diniz Da Costa J.C. (2000). Synthesis and Characterisation of Molecular Sieve Silica (MSS) Membranes. Ph.D. Thesis.

[16] Ellis F.P.K. (2004). Fabrication of Random Hole Optical Fiber Preforms by Silica Sol-Gel Processing. Master’s Thesis.

[17] Elma M., Riskawati N., Marhamah (2018). Silica Membranes for Wetland Saline Water Desalination: Performance and Long Term Stability. Iop Conf. Ser. Earth Environ. Sci..

[18] Elma M., Ayu R., Rampun E.L.A., Annahdliyah S., Suparsih D.R., Sari N.L., Pratomo D.A. (2019). Fabrication of interlayer-free silica-based membranes—Effect of low calcination temperature using an organo-catalyst. Membr. Technol..

[19] Wijaya S., Duke M.C., Diniz da Costa J.C. (2009). Carbonised template silica membranes for desalination. Desalination.

[20] Mir S.H., Nagahara L.A., Thundat T., Mokarian-Tabari P., Furukawa H., Khosla A. (2018). Review—Organic-Inorganic Hybrid Functional Materials: An Integrated Platform for Applied Technologies. J. Electrochem. Soc..

[21] Kanezashi M., Asaeda M. (2006). Hydrogen permeation characteristics and stability of Ni-doped silica membranes in steam at high temperature. J. Membr. Sci..

[22] Darmawan A., Karlina L., Astuti Y., Sriatun, Wang D.K., Motuzas J., da Costa J.C.D. (2017). Interlayer free—Nickel doped silica membranes for desalination. Iop Conf. Ser. Mater. Sci. Eng..

[23] Darmawan A., Karlina L., Astuti Y., Sriatun, Motuzas J., Wang D.K., da Costa J.C.D. (2016). Structural evolution of nickel oxide silica sol-gel for the preparation of interlayer-free membranes. J. Non-Cryst. Solids.

[24] Smart S., Vente J.F., da Costa J.C.D. (2012). High temperature H2/CO2 separation using cobalt oxide silica membranes. Int. J. Hydrog. Energy.

[25] Liu L., Wang D.K., Martens D.L., Smart S., Diniz da Costa J.C. (2015). Interlayer-free microporous cobalt oxide silica membranes via silica seeding sol–gel technique. J. Membr. Sci..

[26] Elma M., Saputro G.S. (2020). Performance of Cobalt-Silica Membranes through Pervaporation Process with Different Feed Solution Concentrations. Mater. Sci. Forum.

[27] Cornelius C.J., Marand E. (2002). Hybrid inorganic–organic materials based on a 6FDA–6FpDA–DABA polyimide and silica: Physical characterization studies. Polymer.

[28] Wang D.K., Elma M., Motuzas J., Hou W.-C., Schmeda-Lopez D.R., Zhang T., Zhang X. (2016). Physicochemical and photocatalytic properties of carbonaceous char and titania composite hollow fibers for wastewater treatment. Carbon.

[29] Rampun E.L.A., Elma M., Syauqiah I., Putra M.D., Rahma A., Pratiwi A.E. (2019). Interlayer-free Silica Pectin Membrane for Wetland Saline Water via Pervaporation. Jurnal Kimia Sains dan Aplikasi.

[30] Pratiwi A.E., Elma M., Rahma A., Rampun E.L.A., Saputro G.S. (2019). Deconvolution of pectin carbonised template silica thin-film: Synthesis and characterisation. Membr. Technol..

[31] Yang H., Elma M., Wang D.K., Motuzas J., da Costa J.C.D. (2017). Interlayer-Free Hybrid Carbon-Silica Membranes for Processing Brackish to Brine Salt Solutions by Pervaporation. J. Membr. Sci..

[32] Elma M., Wang D.K., Yacou C., da Costa J.C.D. (2015). Interlayer-Free P123 Carbonised Template Silica Membranes for Desalination with Reduced Salt Concentration Polarisation. J. Membr. Sci..

[33] Elma M., Wang D.K., Yacou C., da Costa J.C.D. (2015). Interlayer-Free Hybrid Organo-Silica Membranes Based Teos and Tevs for Water Desalination. Conf. Oleo Petrochem. Eng..

[34] Ibrahim S.M., Nagasawa H., Kanezashi M., Tsuru T. (2017). Organosilica bis(triethoxysilyl)ethane (BTESE) membranes for gas permeation (GS) and reverse osmosis (RO): The effect of preparation conditions on structure, and the correlation between gas and liquid permeation properties. J. Membr. Sci..

[35] Wang S., Wang D.K., Jack K.S., Smart S., Diniz da Costa J.C. (2015). Improved hydrothermal stability of silica materials prepared from ethyl silicate 40. Rsc Adv..

[36] Moreth K., Frey H., Hubo M., Zeng-Brouwers J., Nastase M.-V., Hsieh L.T.-H., Haceni R., Pfeilschifter J., Iozzo R.V., Schaefer L. (2014). Biglycan-triggered TLR-2-and TLR-4-signaling exacerbates the pathophysiology of ischemic acute kidney injury. Matrix Biol..

[37] Prakash J., Tripathi B.M., Kumar Ghosh S., Tiwari A., Rawlins J., Hihara L.H. (2015). Chapter 4—Low Temperature Coating Deriving from Metal-Organic Precursors: An Economical and Environmentally Benign Approach. Intelligent Coatings for Corrosion Control.

[38] Elma M., Setyawan H., Rahma A., Pratiwi A.E., Rampun E.L.A. (2019). Fabrication of Interlayer-free P123 Caronised Template Silica Membranes for Water Desalination: Conventional Versus Rapid Thermal Processing (CTP vs RTP) Techniques. Iop Conf. Ser. Mater. Sci. Eng..

[39] Wang S. (2016). High Performance es40-Derived Silica Membranes for Desalination. PhD Thesis.

[40] Liu L., Ding J., Sarrigani G.V., Fitzgerald P., Aljunid Merican Z.M., Lim J.-W., Tseng H.-H., Xie F., Zhang B., Wang D.K. (2020). Enhanced catalyst dispersion and structural control of Co3O4-silica nanocomposites by rapid thermal processing. Appl. Catal. B Environ..

[41] Wang S., Wang D.K., Motuzas J., Smart S., da Costa J.C.D. (2016). Rapid thermal treatment of interlayer-free ethyl silicate 40 derived membranes for desalination. J. Membr. Sci..

[42] Liu L., Wang D.K., Martens D.L., Smart S., Diniz da Costa J.C. (2015). Influence of sol–gel conditioning on the cobalt phase and the hydrothermal stability of cobalt oxide silica membranes. J. Membr. Sci..

[43] Wang D.K., Motuzas J., da Costa J.C.D., Smart S. (2013). Rapid thermal processing of tubular cobalt oxide silica membranes. Int. J. Hydrog. Energy.

[44] Ayu Lestari R., Elma M., Rampun E.L.A., Sumardi A., Paramitha A., Eka Lestari A., Rabiah S., Assyaifi Z.L., Satriaji G. (2020). Functionalization of Si-C Using TEOS (Tetra Ethyl Ortho Silica) as Precursor and Organic Catalyst. E3s Web Conf..

[45] Bonekamp B.C., Burggraaf A.J., Cot L. (1996). Chapter 6: Preparation of asymmetric ceramic membrane supports by dip-coating. Membrane Science and Technology.

[46] Marcos-Hernández M., Villagrán D., Kyzas G.Z., Mitropoulos A.C. (2019). Applications. Composite Nanoadsorbents.

[47] Yoon S.B., Choi B.-S., Lee K.-W., Moon J.-K., Choi Y.S., Kim J.-Y., Cho H., Kim J.H., Kim M.-S., Yu J.-S. (2014). New mesoporous silica/carbon composites by in situ transformation of silica template in carbon/silica nanocomposite. J. Exp. Nanosci..

[48] Thommes M., Kaneko K., Neimark A.V., Olivier J.P., Rodriguez-Reinoso F., Rouquerol J., Sing K.S.J.P., Chemistry A. (2015). Physisorption of gases, with special reference to the evaluation of surface area and pore size distribution (IUPAC Technical Report). Pure Appl. Chem..

[49] Fu L., Zhu J., Huang W., Fang J., Sun X., Wang X., Liao K. (2020). Preparation of Nano-Porous Carbon-Silica Composites and Its Adsorption Capacity to Volatile Organic Compounds. Processes.

[50] Lestari R.A., Elma M., Rahma A., Suparsih D., Anadhliyah S., Sari N.L., Pratomo D.A., Sumardi A., Lestari A.E., Assyaifi Z.L. (2020). Organo Silica Membranes for Wetland Saline Water Desalination: Effect of membranes calcination temperatures. E3s Web Conf..

[51] Mrowiec-Bialon J., Jarzebski A.B., Pajak L., Olejniczak Z., Gibas M. (2004). Preparation and surface properties of low-density gels synthesized using prepolymerized silica precursors. Langmuir.

[52] Mrowiec-Bialon J., Turek W., Jarzębski A. (2002). Preparation of highly active heteropolyacid-silica composite catalysts using the SOL–GEL method. React. Kinet. Catal. Lett..

[53] Jiang H., Zheng Z., Wang X. (2008). Kinetic study of methyltriethoxysilane (MTES) hydrolysis by FTIR spectroscopy under different temperatures and solvents. Vib. Spectrosc..

[54] More P.M., Umbarkar S.B., Dongare M.K. (2016). Template-free sol–gel synthesis of high surface area mesoporous silica based catalysts for esterification of di-carboxylic acids. Comptes Rendus Chim..

[55] Yang H., Wang D.K., Motuzas J., da Costa J.C.D. (2017). Hybrid vinyl silane and P123 template sol−gel derived carbon silica membrane for desalination. J. Sol-Gel Sci. Technol..

[56] Ladewig B.P., Tan Y.H., Lin C.X.C., Ladewig K., Diniz da Costa J.C., Smart S. (2011). Preparation, Characterization and Performance of Templated Silica Membranes in Non-Osmotic Desalination. Materials.

[57] Lin C.X.C., Ding L.P., Smart S., Diniz da Costa J.C. (2012). Cobalt oxide silica membranes for desalination. J. Colloid Interface Sci..

[58] Duke M.C., Mee S., da Costa J.C.D. (2007). Performance of porous inorganic membranes in non-osmotic desalination. Water Res..

[59] Nagarale R.K., Gohil G.S., Shahi V.K., Rangarajan R. (2004). Organic−Inorganic Hybrid Membrane:  Thermally Stable Cation-Exchange Membrane Prepared by the Sol−Gel Method. Macromolecules.

[60] Lee K.P., Arnot T.C., Mattia D. (2011). A review of reverse osmosis membrane materials for desalination—Development to date and future potential. J. Membr. Sci..

